# Paralysis Activity of “Basic Substances” and Rose Extracts on *Meloidogyne incognita* Second-Stage Juveniles

**DOI:** 10.3390/plants15030458

**Published:** 2026-02-02

**Authors:** Rodanthi Askianaki, Nikolaos G. Tsiropoulos, Kyriakos D. Giannoulis, Nikoletta Ntalli

**Affiliations:** 1 Analytical Chemistry and Pesticides Laboratory, Department of Agriculture Crop Production and Rural Environment, University of Thessaly, 38446 Volos, Greece; ntsirop@uth.gr; 2 Laboratory of Agronomy and Applied Crop Physiology, Department of Agriculture, Crop Production and Rural Environment, University of Thessaly, Fytokou Street, 38446 Volos, Greece; kgiannoulis@uth.gr

**Keywords:** rose hydrosol, beer, sodium bicarbonate, sodium chloride, root knot nematodes

## Abstract

To date, searching for bionematicidals is essential. In the absence of nematicides, “Basic Substances” are gaining ground since they are cost-effective, do not mandate an expiration date and have no inherent capacity to cause endocrine-disrupting neurotoxic or immunotoxic effects. Most “Basic Substances” are authorized for the control of phytoparasitic fungi and insects, whereas nematicidals are yet to be available. In this study, we employed “Basic Substances” and in particular, beer, sodium bicarbonate, and sodium chloride, together with rose aromatotherapy by-products, on nematicidal bioassays against *Meloidogyne incognita*. We report that chemical composition analysis of the nematicidal rose extracts correlates with bioactivity. Paralysis-based bioassays were used as primary criteria to assess efficacy, specifically targeting second-stage juveniles of *Meloidogyne incognita*. The evaluated treatments were assessed after one day, two days, and three days of J2 immersion in test solutions. According to our results, the “Basic Substances” demonstrated a significant paralysis effect on J2, thus indicating, for the first time, the considerable significance of their authorization to the root knot nematodes. Similarly, the rose extracts were found to be nematicidal, and since they are foodstuffs, and thus *nonconcern compounds*, “Basic Substances” can be developed as aromatherapy by-products in the frame of a circular economy.

## 1. Introduction

As the criteria for the authorization of Plant Protection Products (PPPs) become increasingly stringent, synthetic substances with greater toxicological concern are gradually being phased out. Consequently, there is a growing need to identify plant protection solutions with a lower environmental impact. In recent years, PPPs of natural origin have emerged as safer alternatives compared to synthetic chemical compounds [1,2]. Τhe term “Basic Substances” refers to certain “products or recipes” whose primary application is not in plant protection, yet they can provide supportive plant protection functions. These substances generally include common food-grade materials, such as sucrose, vegetable oils (e.g., sunflower or onion oil), and non-toxic plant extracts (*Salix* spp. and *Urtica* spp.). By definition, “Basic Substances” are authorized for plant protection, with the prerequisite that they cannot be commercialized. This ensures their cost-effectiveness since the farmer can readily prepare them independently and most importantly use them without a time limitation. These substances gained increasing research attention as potential alternatives to synthetic pesticides, exhibiting fungicidal, bactericidal and insecticidal activity [1,3]. The authorization of the “Basic Substances” is regulated under Article 23 of Regulation (EC) No 1107/2009 of the European Parliament, which governs the authorization of PPPs. Approval prerequisites require that they pose no risk to the environment or to human and animal health at the concentrations used, specifically avoiding endocrine, neurological, or immunological disruptions. As a result, they are not associated with residue-related concerns, and no maximum residue limits (MRLs) are established. Certain “Basic Substances” are also approved as active substances in low-risk PPPs—for example, sodium bicarbonate (baking soda), approved as a low-risk substance, and vinegar, whose primary component, acetic acid, is approved as an herbicide. A notable example is chitosan, which elicits plant defense mechanisms [1,3,4].

The first “Basic Substances” originated from food products, such as vinegar and whey. Subsequently, a hydroalcoholic extract of *Equisetum arvense* was investigated and registered in 2014 for its fungicidal activity. Substances of mineral origin (e.g., talc), animal origin (e.g., chitosan), and microorganisms (e.g., brewer’s or baker’s yeast) have also been registered as “Basic Substances”; as of the latest review (27 December 2025), 28 “Basic Substances” have been approved (Table 1) [5]. Due to their properties, these substances are suitable in organic farming, with 21 specifically authorized for this purpose. They can be applied directly or after dilution; water is the primary diluent used to date, although chitosan has also been approved for dissolution in vinegar. Moreover, the “Basic Substances” are compatible with other plant protection agents, including microorganisms and substances of natural, mineral, or animal origin [1,3,4]. Comprehensive guidelines for their use are provided in Table S1 of Supplementary Materials.

Field efficacy trials of “Basic Substances” against pests and pathogens remain limited. However, the application of hydrochloric chitosan under field conditions has shown effects comparable to certain conventional PPPs [11]. Although the body of scientific literature on “Basic Substances” is still limited, it has been steadily growing in recent years. Their use is considered particularly important within the framework of the European Green Deal’s Farm to Fork strategy [3,4]. While the nematicidal effects of salts and ionic stressors have been widely reported, the present study differs in its practical application focus. Beer, sodium bicarbonate, and sodium chloride are already registered for the control of some agricultural pests, yet their potential against plant-parasitic nematodes has not been evaluated. This regulatory status makes them particularly attractive candidates for timely repurposing in nematode management programs. 

*Rosa damascena*, of the Rosaceae family, is commonly referred to as the Damask rose or the hundred-petaled rose and is recognized as one of the most significant aromatic plant species globally. While frequently cultivated as an ornamental species, its primary value lies in the diverse products obtained from its flowers. The species is widely grown across Asia and Europe, with Bulgaria, Turkey, and Iran being the principal producers.

Botanically, the Damask rose is a perennial, upright shrub that can reach 2.5 m in height, flowering from May to June. The blossoms typically contain around thirty petals, exhibiting vivid shades of pink. A fully mature shrub, aged four years or more, can yield approximately 500 to 600 flowers annually.

Fresh or dried petals, or entire flowers, are utilized to produce essential oil, rose water, extracts, or waxes. These derivatives are employed in the food, fragrance, cosmetic, and pharmaceutical industries. The rose exhibits a range of beneficial properties, including anticancer, antioxidant, and analgesic effects. Additionally, residues from the distillation process are repurposed as animal feed or compost [35]. Toxicological studies on Damask rose extracts have demonstrated a very low risk of toxicity to both animals and humans [36,37] supporting the potential for inclusion in the list of “Basic Substance”.

The primary chemical constituents of the rose include monoterpenic alcohols such as citronellol, geraniol, and nerol; monoterpenes, including rose oxide; secondary metabolites of the shikimate pathway, such as methyl eugenol and phenylethanol; long-chain hydrocarbons; and metabolites derived from carotenoid degradation. Phenylethanol, present in high concentrations in the flowers, contributes to the characteristic aroma of the plant and is readily water-soluble [38].

In the context of exploring naturally derived substances with nematicidal activity, three “Basic Substances” and three rose extracts were selected and assessed for their potential to induce paralysis on *Meloidogyne incognita* second-stage juveniles, the infective stage of the nematode. The “Basic Substances” tested included sodium bicarbonate, sodium chloride, and beer. On the other hand, dried petals from organically cultivated *Rosa damascena* were tested as sonicated water extract and hydrosol, the hydro-distillation byproduct. Chemical analysis of the ultrasound-assisted extract, proved to be the most effective, was conducted to characterize its constituent compounds. To the best of our knowledge these “Basic Substances” along with *Rosa domestica* extracts have not been previously investigated on root knot nematodes.

## 2. Results

### 2.1. Study of Inducing Paralysis of J2 Larvae with Basic Substances

#### 2.1.1. Efficacy of Sodium Bicarbonate

Table 2 lists the EC_50_ values, as calculated for all immersion intervals of the J2 larvae in the sodium bicarbonate solutions, after statistical processing. At 24 h post-immersion, paralysis was induced with an EC_50_ value equal to 15.08 mg/mL. At 48 h, the EC_50_ value decreased to 6.45 mg/mL, and at 72 h, it stabilized at 7.88 mg/mL, at which point the paralyzed larvae were classified as dead, since the paralysis was irreversible.

#### 2.1.2. Efficacy of Sodium Chloride

For all immersion intervals, the EC_50_ values obtained after statistical processing are reported in Table 3. At 24 h post-immersion of J2 larvae in the sodium chloride solutions paralysis was considerable, with an EC_50_ value equal to 7.04 mg/mL. Increasing the duration of immersion to 48 h, resulted in an EC_50_ value equal to 6.47 mg/mL, and 24 h later remained at a similar level (6.45 mg/mL). 72 h later, larvae classified as paralyzed were considered dead.

#### 2.1.3. Efficacy of Beer

Table 4 presents the EC_50_ values calculated for alcohol-free beer solution paralysis on J2 larvae, as determined through statistical analysis. Alcohol-free beer was used to rule out potential toxicity of ethanol. At 24 h post-immersion of J2 larvae in the test solutions, the paralysis activity of the beer was evident, with the EC_50_ value calculated at 3.78% (*v*/*v*). At 48 and 72 h post-experiment establishment, the EC_50_ value was determined at similar levels, 3.35% and 3.07% (*v*/*v*), respectively, and the paralyzed larvae were classified as dead.

### 2.2. Study of Inducing Paralysis of J2 Larvae with Rose Extracts

#### 2.2.1. Efficacy of Rose Petal Extract Produced in Sonicator

Table 5 reports the EC_50_ values calculated after J2 immersion in water extracts of rose petals, produced in the sonicator. The extract induced paralysis and the EC_50_ value was 25.82% (*v*/*v*), 24 h post-J2 immersion in test solutions. Forty-eight hours later, the EC_50_ value decreased to 11.06% *v*/*v*, and seventy-two hours later, the value was set to 2.66% (*v*/*v*), and paralyzed larvae were considered dead. The results presented a positive dose–effectiveness correlation over time.

#### 2.2.2. Efficacy of Rose Petal Hydrosol as Clevenger by Product

Table 6 shows the EC_50_ values calculated after J2 immersion in rose petals’ hydrosol solutions. A clear dose–response relationship was established between induced paralysis over time. At 24, 48 and 72 h post-larvae immersion in test solutions, the EC_50_ value was calculated at 7.73, 5.95 and 4.95% (*v*/*v*), respectively. Motility was not recovered after immersion in water; thus, the larvae were considered dead.

#### 2.2.3. Chemical Analysis of Sonicated Extract of Rose Petals

Table 7 shows the results of the chemical analysis of the rose petals’ extract, with the main components containing (>1%), which constituted 82.2% of the total components. A total of 13.6% of them remained unidentified, while those that were present at <1% in the extract reached a percentage equal to 4.2%.

According to the results, the substances having the highest percentages over total identified compounds were citronellol (21.3%), phenylethyl alcohol (20.7%), cis-geraniol (9.3%), pelargonic acid (6.4%), geranic acid (6.2%), β-citral (3.9%), eugenol (3.5%) and methyleugenol (2.9%).

## 3. Discussion

The risks posed by pesticides constitute a significant problem for society and are related to their residues in the environment and food, with their use being the third greatest concern regarding food safety. For this reason, integrated pest management is essential, which includes the use of natural products as alternatives to synthetically developed industrial formulations [39]. “Basic Substances” and Low-Risk Plant Protection Products containing microorganisms and plant extracts, in addition to direct toxicity on plant parasites, also function as biostimulants, promoting host plant growth and enhancing soil health [40]. They can suppress plant pest development, stimulate the host plant’s defense mechanisms, or promote growth by improving nutrient utilization. Some may not be effective when applied alone against plant pests but are useful when combined with other methods as part of a broader plant protection strategy. Notably, they can be used in organic farming [40]. “Basic Substances” are, by definition, substances that do not raise concern because they do not cause endocrine, neurological, or immunological disorders. They are not classified as plant protection products, but they are useful when applied directly or following dilution. Regarding their approval, compared to low-risk substances, whose approval does not exceed 15 years, “Basic Substances” have no such time limitation and do not have an expiration date [41]. According to Table S1 of Supplementary Materials, the “Basic Substances” approved so far relate to their use against phytopathogenic insects, fungi, bacteria, and viruses or for the disinfection of agricultural tools and have not been approved for nematodes’ control. De Long et al. (2021), for example, in chrysanthemum cultivation, applied hydrochloric chitosan combined with garlic extract, approved as a low-risk nematicidal product in the EU, and demonstrated significant nematicidal action [42]. In this study we investigate the effect, through paralysis experiments, of three “Basic Substances” namely sodium bicarbonate, sodium chloride, and beer.

Sodium bicarbonate is an approved “Basic Substance” for use in the form of a water-soluble powder on vegetables, soft fruits, and ornamental plants to control fungi such as *Sphaerotheca* spp., *Oidium* spp., on grapevines against *Uncinula necator*, and on apple trees against *Venturia inaequalis*. Additionally, it can be used post-harvest on various fruits to combat *Penicillium italicum* and *P. digitatum*, and in dry form on ornamental plants to suppress the growth of thalli such as *Lunularia cruciate* [25].

Other research highlights biological activities, such as combating fungi like *Didymellabryoniae* and *Podosphaera xanthii* in watermelon crops, pests such as *Aphis gossypii*, *Tetranychus urticae*, *Spodoptera exigua*, *S. litura*, and thrips [43]. In a study by Yassen & Sulaiman (2021), the application of 8000 mg/L of sodium bicarbonate solution resulted in a high mortality rate in *Culex pipiens molestus* mosquito larvae, four days after application [44]. Sodium bicarbonate shows synergistic effects when combined with other substances, such as linseed oil, resulting in the rapid immobilization of gregarious desert locusts on the first day of treatment [45].

Regarding sodium chloride, it has been approved as a “Basic Substance” for use in powder form soluble in water in vineyards against the fungus *Erysiphe necator* and as a granular formulation in mushroom cultivation for controlling species of *Cladobotryum* (e.g., *Mycophilum*), *Lecanicillium* (*Verticillium*) *fungicola*, and *Mycogone perniciosa* [21].

Concerning the pesticidal effect of sodium chloride, an example of adding NaCl to the plant nutrient solution resulted in an increase in osmotic potential, which reduced water availability for *Tuta absoluta* larvae, while simultaneously, due to the increased concentration of Na^+^ and Cl^−^ ions in the leaves, it diminished the nutritional capacity of the larvae [46]. In a study of sodium chloride application on soybeans to treat against sucking insects (*Nezaraviridula*, *Piezodorusguildinii*, *Euschistusheros*), it acted as a repellant, and if combined with imidacloprid, it exhibited a synergistic effect [47,48].

The exposure of phytoparasitic nematodes to various salts and, consequently, to the ions they consist of affects their behavior and can cause toxicity. Specifically, *M. javanica* and *M. incognita* are not attracted to ions such as Na^+^ and Cl^−^, while CO_2_ caused irreversible paralysis to *Heterodera* spp. [49]. The presence of ions like Na^+^ or HCO_3_^−^ can influence the parasitic ability of nematodes. The activity of nematodes depends on the concentration of the ions. For example, low concentrations of sodium chloride (NaCl) (0.001 M or 35.5 mg/L) enhanced the activity of *Hirschmanniella oryzae*. Sodium bicarbonate (NaHCO_3_) has been used in a solution of 1000 mg/L to induce paralysis of *M. incognita*. Concentrations of NaHCO_3_ and NaCl above 15,000 mg/L caused significant mortality in *M. incognita* and *M. javanica* larvae [49,50,51].

Beer, as a “Basic Substance”, is approved for use in soil traps as a molluscicide and offers a good alternative to synthetic products due to its high effectiveness and low cost, without burdening the environment [7]. Beer contains volatile compounds that attract snails, such as CO_2_, which regulates their chemoreceptors in the olfactory nerves. In fact, CO_2_ content may also explain beer’s effect on nematodes, as demonstrated by our experimental results. However, the composition of beer can vary, and therefore, its effectiveness can change not only from brand to brand but also between different batches of the same brand [52].

Comparing the effectiveness of sodium chloride (NaCl) and sodium bicarbonate (NaHCO_3_), NaCl appeared more effective with an EC_50_ value of 7 mg/mL at 24 h post-experiment. After two additional days of J2 immersion in test solutions, NaCl and NaHCO_3_ exhibited similar paralysis activity, with EC_50_ values calculated at 6.45 and 7.88 mg/mL. Interestingly the alcohol-free beer caused paralysis at lower concentrations, exhibiting an EC_50_ of 3 mg/mL after 72 h of J2 immersion in test solutions. 

Damask rose is one of the most important aromatic plants in the world. It is used as an ornamental plant, but mainly for the extraction of essential oil from its petals and flowers. During the extraction process, by-products are also utilized, with applications in the food, perfumery, and pharmaceutical industries [38]. In our study, rose extracts, both in the form of petals’ hydrosol and petals’ aqueous extract prepared in a sonicator, demonstrated activity in *M. incognita* paralysis experiments. In particular, the hydrosol was faster in activity since the first day after the establishment of the experiment (ΕC_50_ 7.7% *v*/*v*), while the rose extract prepared in the sonicator followed (EC_50_ 25.8% *v*/*v*). Clear dose and time relationships were established for the sonicated extract and the hydrosol, with the EC_50_ values stabilizing at 72 h post-experiment at 2.6 and 4.9% (*v*/*v*), respectively. Rose petals are rich in polyphenols, specifically flavonols, including kaempferol and quercetin glycosides [53]. These compounds can be obtained both through aqueous extracts and in hydrodistillation by-products [54]. According to the chemical analysis of the dried rose petal extract conducted in our study, the main compounds identified were citronellol, phenylethanol, cis-geraniol, pelargonic acid, geranic acid, *β*-citral, eugenol, and methyleugenol. Among these compounds, geraniol and eugenol, as well as *β*-citral as monoterpenes, have been reported for their nematicidal activity against J2 larvae of *M. incognita*, while geraniol and citral additionally inhibit egg hatching [55].

## 4. Materials and Methods

### 4.1. Development and Maintenance of the M. incognita Population

The initial population of *M. incognita* nematodes was collected in the form of 2nd stage larvae (J2) from infected tomato roots (*Solanum lycopersicum* L.). Approximately 2000 J2 per plant were applied for artificial infection, when the plants had reached the fourth to fifth leaf stage. The infected plants were kept in a chamber under controlled conditions, with a temperature of 25–28 °C, a relative humidity of 60% and a photoperiod of 16 h. Plants were regularly monitored, watered every two to three days, and inspected for other infestations.

#### Method for Collecting Second Stage (J2) Larvae

The roots of the infected plants were monitored for gall formation from the end of the first thirty days onward. When yellow-orange nematode egg sacs appeared on the galls, the plants were selected for root washing. The above-ground parts of the plants were cut and discarded, and the roots were placed in water to remove soil residues. The roots were then cut into 2 cm sections. To collect the J2 larvae, a 4.5% sodium hypochlorite (NaClO) solution in water was used. The roots were immersed in this solution and shaken in a closed container for four minutes. Afterwards, the roots were placed in a double sieve (250 and 38 mesh) and rinsed under running water. From the lower sieve (38 mesh), the eggs released from the disrupted egg sacs were collected using a water extractor and placed in a modified Baermann funnel, to facilitate hatching, which was maintained at approximately 25 °C.

The first collection of this suspension occurred two days after setting up the Baermann funnel. This initial collection was not used in experiments because the resulting larvae were of mixed ages, and there was also the possibility of detecting eggs. The collection process was then repeated every 48 hours, so that the larvae used in experiments were two days old [56].

### 4.2. “Basic Substances” and Rose Extracts

Among the “Basic Substances”, sodium bicarbonate (NaHCO_3_), sodium chloride (NaCl) and non-alcoholic beer were used in paralysis experiments against *M. incognita* J2. Additionally, rose petals (*Rosa damascena*) water extracts produced in a sonicator along with the rose petal hydrosol, a hydro-distillation by-product, were tested on J2 paralysis bioassays. All bioassays were performed twice, and the results are pooled replicates across multiple assays.

### 4.3. Paralysis Bioassays on M. incognita Second Stage (J2) Larvae

The experiments were conducted on 96-well polystyrene plates. The larvae contained in each well were counted using an inverted microscope with a 40× objective lens, and the average number of J2 per well was 15, as reported in our previous studies [2]. The plates were sealed with a lid to avoid evaporation and kept in the dark at room temperature.

A completely randomized block design was implemented, with five treatments and six replicates for each experiment. To prevent contamination of the treatments, due to possible volatility, separate plates were used for each test concentration. The experimental control consisted of larvae immersed in water. After the last motility assessment, at 72 h of J2 immersion in test solutions, the larvae were transferred to plain water after being washed in tap water through a 20 μm pore screen to remove excess test compounds. If paralysis persisted and remained irreversible after additional 24 h, it was recorded as mortality from that point onward and thus individuals were considered dead. This type of mortality assessment has been reported previously [2].

#### 4.3.1. Paralysis Experiments with Basic Substances

##### Preparation of Sodium Bicarbonate and Sodium Chloride Solutions

The test substances were dissolved in water and tested at five concentrations each. The procedure for preparing solutions was the same for both sodium bicarbonate (NaHCO_3_) and sodium chloride (NaCl). In 1 mL of tap water, 40 mg of each substance was dissolved, and the mixture was stirred using a Vortex device until fully homogenized. This was followed by six successive 1:1 (*v*/*v*) ratio dilutions using the same procedure. As a result, concentrations double the desired final levels were obtained, calculated in mg/mL: 40, 20, 10, 5, 2.5, 1.25 and 0.62 (mg/mL). Then, 70 μL of each solution was taken six times, corresponding to the number of repetitions for each treatment, and added to the wells along with the J2 larval suspension in a 1:1 (*v*/*v*) ratio. The final test concentrations of sodium bicarbonate (NaHCO_3_) and sodium chloride (NaCl) in the immersion wells calculated were 20, 10, 5, 2.5, 1.25, 0.62 and 0.31 (mg/mL).

##### Preparation of Beer Solutions

Beer solutions were prepared by dissolving Άλφα^®^ with water by successive dilutions at 1:1 (*v*/*v*). The resulting concentrations were double the intended final concentrations and calculated as a percentage of the original solution (% *v*/*v*): 50, 25, 12.5, 6.25, 3.12, 1.56 and 0.78. Accordingly, 70 μL of each solution was taken six consecutive times and added to wells containing the J2 larval suspension in a 1:1 (*v*/*v*) ratio. Therefore, within the wells, the final desired concentrations, expressed as a percentage of the original solution (% *v*/*v*), were 25, 12.5, 6.25, 3.12, 1.56, 0.78 and 0.39.

#### 4.3.2. Mobility Experiments with Rose Petal Extracts

##### Preparation of Petals Extracts Using Sonicator

Rose petals were first ground using a mill and the plant material was mixed with water at a ratio of 1:20 (*v*/*v*), due to the high water-holding capacity of dried rose. The extraction was performed in an ultrasonic bath for ten minutes at room temperature. Upon completion of the extraction, the mixture was filtered through cotton. This water extract of petals was consecutively diluted six times with water at a 1:1 (*v*/*v*) ratio to prepare solutions of 100, 50, 25, 12.5, 6.25, 3.12, 1.56 and 0.78 (% *v*/*v*). From each solution, 70 μL was added to the treatment well along with 70 μL of J2 larval suspension. The final test concentrations in the treatment well were 50, 25, 12.5, 6.25, 3.12, 1.56, 0.78 and 0.39 (% *v*/*v*).

##### Preparation of Rose Petal Hydrosol Using Hydrodistillation

For the hydrodistillation of essential oil, 22 g of dried petals were initially weighed. The plant samples were placed in spherical flasks with a volume of 1 L, to which 500 mL of tap water was added. The flasks were placed in heating mantles and connected to a Clevenger apparatus. The hydrodistillation process was carried out for three hours. Due to the low yield of rose petals, no essential oil was produced and the hydrosol was used in J2 paralysis bioassays. The hydrosol was filtered from the plant materials and was then tested on J2 at 50, 25, 12.5, 6.25, 3.125, 1.56, 0.78 and 0.39 (% *v*/*v*).

##### Chemical Analysis of Ultrasound Extract of Dried Petals

For the chemical analysis of the rose petal water extract as prepared in sonication, it proved most effective according to paralysis bioassays. 15 g of dried rose petals was used, extracted with 270 mL of tap water in an ultrasonic bath for thirty minutes, without the application of heating. Filtration through cotton was performed, and 150 mL of extract was collected. To obtain the organic components and separate them from the aqueous solution, extraction with 10 mL of hexane in a separatory funnel followed. Then, 5 mL of the hexane extract was collected, concentrated to a final volume of 1.5 mL, and finally analyzed using a gas chromatography–mass spectrometry system. Separation and determination were performed using an Agilent 6890N GC (Agilent Technologies, Santa Clara, CA, USA) equipped with an Agilent 7683B Injector (Agilent Technologies, Santa Clara, CA, USA), a 30 m, 0.25 m.m i.d, HP-5MS capillary column (Agilent Technologies, Santa Clara, CA, USA) coated with 5% Phenyl-methylpolysiloxane (film thickness) 0.25 μm and an Agilent 5975 mass selective detector (MSD). The samples were injected in the pulsed splitless mode at an injection temperature of 280 °C. Mass spectrometry was acquired using the electron ionization (EI). The chromatograms were processed using appropriate software (Agilent, ChemStation LC B.04.03), while the identification was carried out according to the Total Ion Current (TIC) scan spectrum and compared with standard solutions and the NIST spectral library.

### 4.4. J2 Paralysis Evaluation

The paralysis of J2 larvae was assessed on an inverted microscope at 40× magnification and they were divided into two categories, mobile and paralyzed. Assessments of paralysis were carried out at 24, 48, and 72 h post-J2 immersion in test solutions. Throughout the experiments, the plates were maintained at approximately 25 °C in the dark.

### 4.5. Statistical Analysis

For the processing of paralysis results, the percentages of paralysis were calculated for each treatment, and the natural mortality of the control was removed according to Schneider-Orelli’s formula: Increase in paralysis % = {(paralysis % in the treatment − paralysis % in the control)/(100 − paralysis % in the control)} × 100 [57]. Data from experimental replicates in time were analyzed (ANOVA) after being combined over time. As ANOVA indicated no significant treatment by time interaction, means were averaged over experiments. Corrected percentages of paralyzed J2 treated with rose extracts or “Basic Substances” were subjected to non-linear regression analysis. Data were analyzed using the SPSS program v30 in an analysis of variance (ANOVA) and applied to the log-logistic equation [58] to calculate EC_50_ values.

y = C + (D − C)/[1 + (x/EC_50_)^b^]

where C = the lower limit, D = the upper limit, b = the slope of the line at the EC_50_ value, and EC_50_ = the concentration of the test substance(s) required for a 50% increase in paralyzed J2 compared to the control. In this dose–response equation, the concentration of the extract (μg/mL) was the independent factor (x) and the immobilized J2 (percentage increase over the control) was the dependent factor (y) [58].

## 5. Conclusions

In summary, the substances that were applied induced paralysis in *M. incognita* nematodes. Among “Basic Substances”, sodium bicarbonate and sodium chloride showed similar effects and were more effective at lower concentrations compared to non-alcoholic beer. The presence of Na^+^, Cl^−^ or HCO_3_^−^ ions in the control solutions likely contributed to paralysis in J2 larvae. Although “Basic Substances” have not been approved for use against nematodes, they could be further investigated for their potential nematicidal activity. On the other hand, the rose petal extracts could be of interest for development into “Basic Substances” since they are foodstuffs and thus of *no concern*. In particular, the hydrosol used as a plant protectant could play a role in circular economy too, since it is a hydrodistillation by-product. Nonetheless, although the tested substances have clear potential and practical value in sustainable and integrated field applications, their use under real field conditions might be constrained by variability, limited stability, and formulation challenges. Variability in beer and rose material may result in fluctuations in extract composition and potency, while environmental factors can rapidly reduce stability and efficacy. Limited standardization, formulation, and scalability of the “Basic Substances” may further impact reproducibility and shelf life, thus requiring frequent applications and higher costs, alongside complex regulatory requirements despite their natural origin. On the other hand, although irreversible paralysis is a practical and widely applied endpoint in in vitro assays, it does not necessarily reflect true mortality, as juveniles that appear irreversibly paralyzed under laboratory conditions may retain residual metabolic activity or regain functionality under more favorable environments, such as soil or in association with host roots. To address this limitation, paralysis-based mortality estimates are usually supplemented with additional endpoints like infectivity assays on host plants. We are currently performing pot bioassays with the tested substances presented herein, so as to evaluate effects on nematode life cycle completion in tomato root hosts and thereby link in vitro findings to biologically relevant outcomes under field-relevant conditions.

## Figures and Tables

**Table 1:** “Basic Substances” approved for crop protection in the EU as of the latest review, 27 Dec 2025.

No.	Basic Substances	Target Pests	Source
1	*Allium cepa* L. bulb (extract)	Plant Pathogens	[6]
2	*Allium fistulosum* (processed)	Plant Pathogens	[7]
3	Beer	Pest Slugs and Snails	[8]
4	Calcium Hydroxide	Plant Pathogens	[9]
5	Chitosan	Plant Elicitor Against Plant Pathogens	[10]
6	Chitosan Hydrochloride	Plant Elicitor Against Plant Pathogens	[11]
7	Clayed Charcoal	Plant Pathogens	[12]
8	Cow Milk	Plant Pathogens	[13]
9	Diammonium Phosphate	Plant Pathogens	[14]
10	*Equisetum arvense* L.	Plant Pathogens and Insect Pests	[15]
11	Fructose	Plant Pathogens and Insect Pests	[16]
12	Hydrogen Peroxide	Plant Pathogens	[17]
13	L-cysteine	Leaf Cutting Ants	[18]
14	Lecithins	Plant Pathogens	[19]
15	Magnesium hydroxide E528	Plant Pathogens	[20]
16	Mustard Seeds Powder	Plant Pathogens	[21]
17	Sodium Cloride	Plant Pathogens	[22]
18	Onion Oil	Insect Pests	[23]
19	*Onobrychis viciifolia* (sainfoin) dried pellets	Phytoparasitic Nematodes	[24]
20	*Salix* spp. Cortex	Plant Pathogens	[25]
21	Sodium Hydrogen Carbonate	Plant Pathogens	[26]
22	Sucrose	Plant Pathogens and Insect Pests	[27]
23	Sunflower Oil	Plant Pathogens	[28,29]
24	Talc	Plant Pathogens and Insect Pests	[30]
25	*Urtica* spp.	Insect Pests	[31]
26	Vinegar	Plant Pathogens	[32]
27	*Vitis vinifera* L. seed extract (grape seed extract)	Plant Pathogens	[33]
28	Whey	Plant Pathogens	[34]

**Table 2:** EC_50_ values and corresponding confidence limits, 24, 48 and 72 h after the immersion of *M. incognita* J2, in aqueous solutions of sodium bicarbonate.

Duration of Immersion (NaHCO_3_)	R^2^	EC_50_ (mg/mL)	95% Confidence Limits	Max Efficacy (%)	Min Efficacy (%)	Natural Paralysis (%)
24 h	0.93	15.08	13.83–16.33	84	0	7
48 h	0.79	6.45	5.05–7.84	90	21	12
72 h	0.80	7.88	6.39–9.36	94	8	12

**Table 3:** EC_50_ values and corresponding confidence limits, 24, 48 and 72 h after the immersion of *M. incognita* J2, in aqueous solutions of sodium chloride.

Duration of Immersion (NaCl)	R^2^	EC_50_ (mg/mL)	95% Confidence Limits	Max Efficacy (%)	Min Efficacy (%)	Natural Paralysis (%)
24 h	0.88	7.04	5.98–8.11	89	6	7
48 h	0.79	6.47	5.06–7.87	94	11	12
72 h	0.79	6.45	5.05–7.84	95	11	12

**Table 4:** EC_50_ values and corresponding confidence limits, 24, 48 and 72 h after the immersion of *M. incognita* J2, in aqueous solutions of alcohol-free beer concentrations.

Duration of Immersion (Alcohol-Free Beer)	R^2^	EC_50_ % (*v*/*v*)	95% Confidence Limits	Max Efficacy (%)	Min Efficacy (%)	Natural Paralysis (%)
24 h	0.97	3.78	3.62–3.94	94	6	19
48 h	0.70	3.35	2.47–4.23	100	7	25
72 h	0.85	3.07	2.60–3.53	82	0	30

**Table 5:** EC_50_ values and corresponding confidence limits, 24, 48 and 72 h after the immersion of *M. incognita* J2, in test solutions of dried rose petals water extract produced in sonicator.

Duration of Immersion (Sonicated Rose Petals in Water)	R^2^	EC_50_ % (*v*/*v*)	95% Confidence Limits	Max Efficacy (%)	Min Efficacy (%)	Natural Paralysis (%)
24 h	0.72	25.82	20.4–31.2	100	1	16
48 h	0.85	11.06	9.09–13.02	100	0	25
72 h	0.74	2.66	1.18–4.13	100	62	33

**Table 6:** EC_50_ values and corresponding confidence limits, after 24, 48 and 72 h of *M. incognita* J2 immersion in hydrosol solutions of rose petals.

Duration of Immersion (Rose Petals Hydrosol)	R^2^	EC_50_ % (*v*/*v*)	95% Confidence Limits	Max Efficacy (%)	Min Efficacy (%)	Natural Paralysis (%)
24 h	0.82	7.73	6.27–9.18	80	0	23
48 h	0.75	5.95	3.69–8.21	87	33	25
72 h	0.74	4.94	3.80–6.07	91	29	24

**Table 7:** The major constituents (>1%) as determined by chemical analysis of dried rose petals extracted with water in sonicator.

Spectra Database NIST	Retention Time (Rt)	Substance Content (%) over Total Identified
4-methylhexan-2-one	4.358	2.8
2-hexanone, 3.4-dimethyl-	4.809	1.0
phenylethylalcohol/Rose Oil	12.485	20.7
rose oxide	13.281	1.4
citronellol/rhodinol	19.161	21.3
*β*-citral	19.704	3.9
*cis*-geraniol	20.545	9.3
pelargic acid	21.310	6.4
carvacrol	22.946	1.4
eugenol	25.523	3.5
geranic acid/neric acid	26.028	6.2
*n*-decanoic acid	26.510	1.4
methyl eugenol	27.840	2.9

## Data Availability

The original contributions presented in this study are included in the article. Further inquiries can be directed to the corresponding authors.

## Supplementary Materials

The following supporting information can be downloaded at: https://www.mdpi.com/article/10.3390/plants15030458/s1, Table S1: “Basic Substances” authorized in the EU as at the date 25 December 2025.
