# Supplementary material for: Paralysis Activity of “Basic Substances” and Rose Extracts on Meloidogyne incognita Second-Stage Juveniles
Source: Plants (Basel). 2026 Feb 2;15(3):458. doi: 10.3390/plants15030458 (PMC12899623; doi:10.3390/plants15030458)
Supplement: Supplementary file 1 [file plants-15-00458-s001.zip › plants-4098949-supplementary.pdf]

Supplementary Materials

Table S1. "Basic Substances" authorized in the EU as at the date 25 December 2025.

No	Basic Substances	Pests or group of pests controlled	Crop and/ or situation	Type	Formulation	Application	Water (l/ha) min-max	No. of Application min-max	PHI (days)	Source
1	*Allium cepa* L. bulb (extract)	*Alternaria solani*	Potato	Dispersible concentrate (DC)	Extract 50g *Allium cepa* L. bulb/ L of water	10 g of extract/ L of water	600-1000	3-5 per year	7	[6]
		Phytophthora infestans	Tomato				1500		3-4	
		Botrytis cinerea	Cucumber						7	
2	*Allium fistulosum* (processed)	*Ralstonia solanacearum*	Tomato	Dispersible concentrate (DC)	500 g dried *A. fistulosum* /L	50 kg *A. fistulosum* /hl	650-750	1 per crop =1-3 per year	N/A	[7]
				Plant rodlet (PR)	Raw *A. fistulosum* plant	250g of raw *A. fistulosum* / 1kg soil	0			
3	Beer	Pest slugs and snails	All edible and non-edible crops	AL Other liquids to be applied undiluted	Pure	Specific traps for slugs, 1 trap/ m²	-	1-5 per year	-	[8]
4	Calcium Hydroxide	*Neonectria galligena*	Pome fruit	Liquid suspension (acqueous)	With products at 24%	a) 104-208 l/ha b) 1460 l/ha	5000-10000	2-7	5-14	[9]
		Neonectria galligena	Pome fruit and stone fruit		With products at 24% or 33.12%	products at 24% a) 63-104 l/ha				

						b) 728 l/ha<br><br>products at 33.12%<br>a) 45 – 76 l/ha<br>b) 532 l/ha				
						Brush application directly on pruning wounds and old cancers on stems	-	1-2	21	
5	Chitosan:	Plant elicitor of plant resistance against pathogenic fungi and bacteria	Olive trees	Soluble Powder (SP)	≥ 85% Chitosan	50-100 g/hl	200-400	4-8 per year	14	[10]
			Grapevine				200-600			
			Grass				200-400			
			Ornamentals plants							
			Post-harvest fruit treatment			Immersion	-	1 per year	-	
			Fruits berries and small fruit			50-100 g/hl	200-400	4-8 per year	14	
			Vegetables			50-100 g/hl				
			Cereals	Soluble Powder (SP)	≥ 85% Chitosan	50-100 g/hl	200-400	4-8 per year	14	
			Spices							

			Crops for animal feed							
			Cereals Seed treatment				-	1 per year	-	
			Potato Seed treatment				-		-	
			Sugar beet Seed treatment			50 - 200 g/hl	-		-	
6	Chitosan Hydrochloride	Plant elicitor of plant resistance against pathogenic fungi and bacteria	Grapevine	Soluble Powder (SP)	100% Chitosan Hydrochloride	50 - 200 g/hl	200-400	4-8 per year	14	[11]
						50-100 g/hl	200-600			
			Ornamental Plants				200-400			
			Vegetables							
			Cereals							
			Spices							
			Crops for animal feed							
			Cereals Seed Treatment							
			Potato							
			Sugar beet			50 - 200 g/hl	-	1 per year	-	
						50-100 g/hl	-		-	

			Ornamental herbaceous plants - bulbous plants			50 - 200 g/hl	-		-	
			Arable crops - Beet crops				200-400		-	
7	Clayed Charcoal	*Phaeoacremonium* spp.	Grapevine	Granule (GR)	Mixture of charcoal, meeting the criteria of the food additive E 153 (vegetable carbon), and bentonite, meeting the criteria of feed additive E 558, in the form of granules.	Soil Burying: 50 kg/ha	-	1 every 3 years	-	[12]
8	Cow Milk	*Erysiphe necator*	Grapevine	Soluble Concentrate(SL)	100% Cow Milk	10-40 L/hl	100-300	3-6 per year	6-8	[13]
		Podosphaera xanthii	Vegetable Gardeding Pumpkin (*Cucurbita pepo*)			50 L/hl	400	3-4 per year	7-12	

		Erysiphe cichoracearum	Flower Gerbera (*Gerbera jamesonii*)			16 L/hl	500-1000		7	
		Sphaerotheca fuliginea	Cucumber	Soluble Concentrate (SL)	100% Cow Milk	5-10 L/hl	1000-1500	3-4 per year	7	
		Erysiphe diffusa	Soybean (*Glycine max*)			18 L/hl				
		Viruses	Glove fingertips and mechanical cutting tools All crops			Dipping	-	-	-	
9	Diammonium Phosphate	*Ceratitis capitata, Rhagoletis cerasi*	Cherry	Vapour releasing product (VP)	40g Diammonium Phosphate/ L water	Mass trapping: 1 trap per tree up to 100 traps/ha	-	-	42-56	[14]
		Ceratitis capitata	Other crops where *C. capitata* cause damage							
10	*Equisetum arvense* L.	*Venturia inaequalis, Podosphaera leucotricha, Taphrina deformans*	Apple Peach	Dispersible concentrate (DC)	Extract: 2g *Equisetum arvense* L./L of water	200 g/hl	500-1000	2-6	7	[15]
		Plasmopara viticola, Erysiphe necator	Grapevine				100-300			
		Podosphaera xhantii, Pythium spp.	Cucumber				300	2	3-4	

				Dry (D)	Dry Plant aerial parts	9 kg Included in mulch/ha	-	1	-	
		Alternaria solani, Septoria lycopsersici	Tomato	Dispersible concentrate (DC)	The theoretical concentration of aerial part dry plant present in the decoction is 20 g/L, which is then diluted by 10, hence 2 g/L in the final preparation applied on plants.	2 g/ L water	300	2	14	
				Dry (D)	Dry Plant aerial parts	9 kg Included in mulch/ha	-	1	-	

		Botrytis cinerea, Podosphaera aphanis, Phytophthora fragariae, Colletotrichum acutatum	Strawberry Raspberry	Dispersible concentrate (DC)	The theoretical concentration of aerial part dry plant present in the decoction is 22,5 g/L, which is then diluted by 10, hence around 2,25 g/L in the final preparation applied on plants.	2,25 g/ L water	300	4-8	5-14	

	Equisetum arvense L.	*Phytophthora infestans, Alternaria solani, Erysiphe cichoracearum*	Potato	Dispersible concentrate (DC)	The theoretical concentration of aerial part dry plant present in the decoction is 22,5 g/L, which is then diluted by 10, hence around 2,25 g/L in the final preparation applied on plants.	2,25 g/ L νερού	300	4-8	5-14	
		Marsonia spp., *Phragmidium mucronatum, Oidium* spp.	Ornamental Trees	Dry (D)	Dry Plant aerial parts	9 kg Included in mulch/ha	-	1	-	
11	Fructose	*Cydia pomonella*	Apple	Soluble Powder (SP)		10 g/hl	60-100	5-7	21	

		Scutigerella immaculata	Maize		Pure Fructose: 99.8-100%		40-82	1-2	7	European Commission, 2020c
		Scaphoïdeus titanus	Grapevine				150	3	7	
		Plasmopara viticola					100-200	12	15	
12	Hydrogen Peroxide	*Ralstonia solanacerum, Botrytis cinerea*	Agricultural mechanical cutting tools for *Solanaceae* species	-	Pure: <5% Hydrogen Peroxide	Immersion: 15-30 g hydrogen peroxide solution/L	-	-	-	[17]
		Xanthomonas campestris pv. vitian	Lettuce	Liquid for Seed Treatment (LS)		10-15 g/L	-	1	-	
		Alternaria zinnia, Alternaria alternata, Fusarium spp.	Horticulture flowers			25-49 g/L	-	1	-	
13	L-cysteine	Leaf cutting ants	All crops and forestry in tropical areas	Bait, Ready for use (RB)	5-80 g/kg L-cysteine in matrix (wheat flour)	Hand-held spreader 3 - 36 kg of granules/ha	-	1-3	30	[18]
14		*Podosphaera leucotricha, Taphrina deformans*	Apple Peach	Emulsifiable Concentrate (EC)	Pure Lecithins: 99-103%	75 g/hl	500-1000	3-12	5	[19]

	Lecithins	*Microsphaera grossulariae*	Gooseberry (*Ribes uva-crispa)*	Emulsifiable Concentrate (EC)	Pure Lecithins: 99-103%	200 g/hl	500-1000	2-4	5	European Commission, 2018a
		Podosphaera xhantii	Market vegetables			150 g/hl	1000-1500	2-6		
		Erysiphe cichoracearum	Lettuce					2	7	
		Erysiphe polyphaga	*Mash (Valerianella locusta)*					1	-	
		Phytophthora infestans	Tomato					2-6	7	
		Alternaria cichorii	*Endive (Cichorium endivia* L.)							
		Oidium spp.	Ornamentals			75 g/hl	100-300	3-12	5	
		Plasmopara viticola, Erysiphe necator	Grapevine							
		Podosphaera aphanis, Phytophthora fragariae	Strawberry Raspberry			200 g/hl	300-500			
		Phytophthora infestans	Potato				100-400			

15	Magnesium hydroxide E528	*Plasmopara viticola, Erysiphe necator, Guignardia bidwellii*	Grapevine	Suspension Concentrate (S C)	Suspension Concentrate 700 g/L	437-3500 g/hl	100-800	1	0	[20]
		Cycloconium oleaginum	Olives			700-2330 g/hl	300-1000	1		
		Mycosphaerella spp.	Banana			1600-7000 g/hl	80-350	12		
		Septoria spp.	Cereals Oat, Rye, Triticale, Wheat			160-700 g/hl	80-350	3		
		Phytophtora infestans	Tomato, aubergine, sweet pepper, chilli, *Physalis* sp., Pepino			560-930 g/hl	600-1000	3		
		Phytophtora infestans	Potato			840-1400 g/hl	300-500	10		
		Sphaerotheca pannosa	Rose-Bush			290-580 g/hl	600-1200	2		
		Sphaerotheca pannosa	Ornamental plants			290-580 g/hl	600-1200	2		
		Sphaerotheca pannosa	Stone fruits			290-580 g/hl	600-1200	2		
		Magnaporthe grisea	Rice			1600-7000 g/hl	80-350	3		
16	Mustard Seeds Powder	*Tilletia caries, T. foetida*	Wheat	Water dispersible powder for slurry seed	Mix 1.5 kg of mustard seeds powder	Treat 100 kg seeds with the	-	1	-	[21]

				treatment (WS)	with 4.5 L ofwater.	slurry created.				
17	Sodium Cloride	*Erysiphe necator*	Grapevine	Soluble Powder (SP)	Pure Sodium Chloride: 97%	600-2000 g/hl	200	1-2	-	[22]
		Fungal diseases like cobweb disease Cladobotryum strains (i.e. *Mycophilum*); Dry Bubble Disease *Lecanicillium* (*Verticillium*) fungicola Wet bubble disease Mycogone perniciosa	Mushrooms (*Agaricus bisporus)*	Granules (GR)		0.03g/kg of substrate	-	1		
18	Onion Oil	*Psila rosae*	Carrots Celeriac Parsnip Parsley	Oil Dispension (OD)	Pure Onion Oil	Pot Dispenser 0.08-0.160 L/ha<br><br>Granule Dispenser 17.6 – 35.2 g/ha	-	1	-	[23]

19	*Onobrychis viciifolia* (sainfoin) dried pellets	*Xiphinema index*	Grapevine	Granules (GR)	Dried pellets	Manure spreading Soil incorporation Autumn or Spring 10000 kg/ha<br><br>On disease spots 1,000 g/m2	-	1	-	[24]
20	*Salix* spp. Cortex	*Taphrina deformans*	Peach	Dispersible concentrate (DC)	Extract: 2.22g Salix spp./L	222.22g/hl	500-1000	2-6	7	[25]
		Venturia inaequalis, Podosphaera leucotricha	Apple							
		Plasmopara viticola, Erysiphe necator	Grapevine				100-300			
21	Sodium Hydrogen Carbonate	*Sphaerotheca* spp., *Oidium* spp.	Vegetables Soft fruit Ornamentals	Soluble Powder (SP)	Pure Sodium Hydrogen Carbonate : 99%	333-1000 g/hl	300-600	1-8	10	[26]
		Uncinula necator	Vine			420-2000 g/hl	200-600			

		Venturia inaequali	Apple			500-1000 g/hl	500-1000			
		Penicillium italicum, P. digitatum	oranges, cherries, apples, papaya			1000-4000 g/hl	-	1-2		
		Lunularia cruciata	Potted Plants	Dry (D)		Direct application of powder: 122 kg/ha	-	1	-	
22	Sucrose	*Cydia pomonella*	Apple	Soluble Powder (SP)	Pure Sucrose: 99.8-100%	10 g/hl	600-1000	7-10	15	[27]
		Ostrinia nubilalis	Sweet Maize				200	3-4		
		Ostrinia nubilalis	Maize							
		Scaphoideus titanus	Grapevine				150	3	7	
		Plasmopara viticola					100-200	Up to 12		
23	Sunflower Oil	*Oidium neolycopersici*	Tomato	Oil Dispension (OD)	Pure Sunflower Oil : 91.5-92.3%	0.092 (0.1L)-0.46 (0.5L)/hl	500-1000	2-4	8	[28,29]
		Uromyces appendiculatu	Bean		Pure Sunflower	0.092 (0.1L)/hl	200-500	1-3	7	

		Podosphaera xanth	Cucumber	Oil Dispension (OD)	Oil: 91.5-92.3%			1-5		
		Podosphaera spp.	*Rosaceae* Species				200-700	1-7		
		Podosphaera leucotricha	Apple<br>Pear				200-600			
		Plasmopara viticola	Grapevine				200-300			
		Puccinia triticina, P. hordei	Wheat<br>Barley				100-300	1-3		
		Phytophthora infestans	Potato					1-7		
		Alternaria dauci	Carrot			0.46 (0.5L)/hl	200-500	1-3	15	
24	Talc	*Cacopsylla pyri, Cacopsylla fulguralis, Drosophila suzukii, Panonychus ulmi, Bactrocera oleae*	Fruit Trees (*Malus domestica, Pyrus* sp., *Olea europea* κλπ)	Wettable Powder (WP)	Pure Talc : 85%	2.13-3.54 kg/hl	600-1000	2-5	21-28	[30]
		Venturia inaequalis, Erysiphe necator				1.28-2.13 kg/hl		3-5	14-21	

		Venturia inaequalis, Erysiphe necator	Grapevine			4.25-8.5 kg/hl	150-300	2-5	21-28	
25	*Urtica* spp.	*Myzus persicae, Macrosiphum rosae, Eriosoma lanigerum, Cryptomyzus ribis, Callaphis juglandi, Myzus cerasi*	Apple Plum Peach Redcurrant Walnut Cherry	Dispersible concentrate (DC)	Up to 75g/L (fresh nettle) Or 15g/L (dry matter) Filtration	1500g/hl (dry matter)	300-900	1-5	7-15	[31]
		Aphis fabae	Bean				300-500			
		Myzus persicae	Potato				300-500			
		Brevicoryne brassicae, Nazonovia ribisnigri	Lettuce Cabbage							
		Aphis sambuci	Elder Tree (*Sambucus racemosa*)				400-800			
		Macrosyphum rosae	Rose				300-600			
		Aphis spiraephaga	*Spiraea* sp.				300-500	1-6		
		Phyllotreta nemorum								

		Plutella xylostella	Cabbage rapeseed radish							
		Cydia pomonella	Apple Peer				300-900	3	15	
		Tetranychus urticae	Bean				300-500	1-6	7-21	
		Tetranychus urticae	Grapevine				300-600			
26	Vinegar	*Tilletia caries, T. foetida*	Wheat	Liquid for Seed Treatment (LS)	1/1 dilution of vinegar/water L/L	25-50 g per 100 kg of Seed	-	1	-	[32]
		Pyrenophora graminea	Barley				-		-	
		Alternaria spp.	Market vegetables	Liquid for Seed	1/1 dilution of	25-50 g per 100 kg of Seed: Seeds	-	1	-	

		Clavibacter michiganensis, Clavibacter michiganensis subsp. michiganensis, Pseudomonas syringae pv. Tomato, Xanthomonas campestris pv. Vesicatoria, Botrytis aclada		Treatment (LS)	vinegar/wat er L/L	are temporary soaked in the dilution then removed				
		Pseudomonas syringae pv aesculi	*Aesculus* L., *Sycamore* spp., *Acer* spp.	Liquid for disinfection of mechanical cutting tools (LS)	50ml/1 L dilution of vinegar/wat er for vinegar at 8% acetic acid	Tools application before sawing or cutting Waiting period 30 seconds after washing				
		Erwinia amylovora	Hawthorns (*Rosaceae)*							
		Pseudomonas syringae pv. syringae	Medicinal aromatic and perfume crops							
		Phellins phellinus,Tinder polypore, Fomes fomentarius	*Plane* sp., *Platanus, Prunus* sp., *Chestnut* sp., *Aesculus L. Sophora* spp., *Tilia* spp.							
		Ophiostoma spp.	*Ulmus* spp.							

		Verticillium spp.	*Maple* sp., *Ailanthe* sp., *Ailanthus altissima*							
		Cryptostroma corticale	*Maple* sp., *Acer* sp., *Sycamore, Acer* spp., *Chestnut* sp., *Aesculus L., Beech* sp., *Fagus* spp.							
27	*Vitis vinifera* L. seed extract (grape seed extract)	*Plasmopara viticola*	Grapevine	Wettable Powder (WP) or Dustable Powder (DP)	100 % grape seed powder	98 g/hl	300	6	-	[33]
		Venturia inaequalis	*Apple*				500	6		
		Phytophthora infestans	Potatoes				300	6		
		Peronospora sp., *Bremia lactucae*	Lettuce				500	3		

28	Whey	*Podosphaera fusca, Podosphaera xanthii, Golovinomyces/Erysiphe cichoracearum, E. orontii, Sphaerotheca fuliginea, Leveillula cucurbitacearum*	Cucumber Zucchini squash	Technical Material (TC)	60-80 g/L water	0.24 g/ L water	1000-1500	3-5	7	[34]
		Erysiphe necator	Grapevine			0.36-2.4 g/ L water	100-300		7-10	
		Tomato (Sinaloa) yellow leaf curl virus (*Begomovirus*)	Tomato			0.036-0.24 g/ L water	1000-1500		3-4	
		Tobacco mosaic virus (TMV), Tomato mossaic virus (ToMV), Pepper mild mottle virus (PMMV), Cucumber green mottle mosaic virus (CGMMV), Tomato brown rugose fruit viruses (ToBRFV)	Glove fingertips and mechanical cutting tools All crops		50 g/L water	Dipping	-	-	-	
